# Members of Glycosyl-Hydrolase Family 17 of *A. fumigatus* Differentially Affect Morphogenesis

**DOI:** 10.3390/jof4010018

**Published:** 2018-01-30

**Authors:** Nicolas Millet, Jean-Paul Latgé, Isabelle Mouyna

**Affiliations:** Aspergillus Unit, Institut Pasteur, 25 rue du Docteur Roux, 75015 Paris, France; nicolas.millet@pasteur.fr (N.M.); jplatge@pasteur.fr (J.-P.L.)

**Keywords:** *Aspergillus fumigatus*, cell wall remodeling, Glycosyl-Hydrolase family 17, glucanase, β(1-3)glucanosyltransferase

## Abstract

Cell wall biosynthesis and remodeling are essential for fungal growth and development. In the fungal pathogen *Aspergillus fumigatus*, the β(1,3)glucan is the major cell wall polysaccharide. This polymer is synthesized at the plasma membrane by a transmembrane complex, then released into the parietal space to be remodeled by enzymes, and finally incorporated into the pre-existing cell wall. In the Glycosyl-Hydrolases family 17 (GH17) of *A. fumigatus*, two β(1,3)glucanosyltransferases, Bgt1p and Bgt2p, have been previously characterized. Disruption of *BGT1* and *BGT2* did not result in a phenotype, but sequence comparison and hydrophobic cluster analysis showed that three other genes in *A. fumigatus* belong to the GH17 family, *SCW4*, *SCW11*, and *BGT3*. In constrast to *Δbgt1bgt2* mutants, single and multiple deletion of *SCW4*, *SCW11*, and *BGT3* showed a decrease in conidiation associated with a higher conidial mortality and an abnormal conidial shape. Moreover, mycelium was also affected with a slower growth, stronger sensitivity to cell wall disturbing agents, and altered cell wall composition. Finally, the synthetic interactions between Bgt1p, Bgt2p, and the three other members, which support a functional cooperation in cell-wall assembly, were analyzed. Our data suggest that Scw4p, Scw11p, and Bgt3p are essential for cell wall integrity and might have antagonistic and distinct functions to Bgt1p and Bgt2p.

## 1. Introduction

*Aspergillus fumigatus* is the most common airborne fungal pathogen, causing fatal invasive aspergillosis in immunocompromised patients [1]. One of the most important characteristics of the fungal cell is the presence of an outer and rigid layer called the cell wall, which surrounds it. This biological coat is both a rigid and protective exoskeleton and a dynamic structure. In *A. fumigatus*, as in other pathogenic fungi, the cell wall acts as a sieve and reservoir of antigens, enzymes, and toxins that play an active role during infection. Essential for growth, it provides the organism with a resistance from the internal turgor pressure, and protects the cell from its environment (e.g., desiccation and osmotic stress) [2,3]. Moreover, the cell wall is in contact with the external environment and therefore plays an important role in the interaction between the pathogen and the host [4,5]. Because of its absence in humans and its essentiality, the fungal cell wall is a promising target for antifungal drugs and therefore, it is important to elucidate cell wall biosynthesis and organization.

The cell wall of *A. fumigatus* accounts for about 30% of the cell dry weight and is mainly constituted of different polysaccharides (at least 90%) [6,7]. β(1,3)glucan (glucose polymer bound with β(1,3) bonds) is the main component of the *A. fumigatus* cell wall. β(1,3)glucan chains are connected together by β(1,6) linkages (4% of branching points), forming a three-dimensional network. It constitutes the primary skeleton of the fungal wall, on which other polysaccharides are covalently linked (e.g., Chitin, Galactomannan). Thus, crosslinking of the β(1,3)glucan chains ensures the rigidity of the wall, which is essential for the survival and growth of the fungus.

During the various stages of the fungal life, the cell wall is a dynamic structure, which is constantly reorganized. The continuous processes of synthesis and degradation of the parietal material are highly regulated in order to ensure the biological functions of the cell wall. β(1,3)glucan is synthesized as a linear polymer by a plasma membrane-bound synthase complex using UDP-glucose as a substrate and extruded as linear β(1-3)glucan chains through the membrane into the periplasmic space [8,9]. Upon arrival in the cell wall space, its remodeling involves extracellular enzymatic activities such as β(1,3)glucanases and transglucosidases, which are known to play an important role in the modification of cell wall glucan [10]. Glycoside-Hydrolases (GH) have been classified by sequence and structure homology within the CAZy (Carbohydrate-Active enZYmes, [11]) database [12]. Several GH families have already been characterized and play an important role in the architecture of the fungal cell wall [13].

The first family of β(1,3)glucanosyltransferase described in *A. fumigatus* has been the *GEL* family (GH72) which consists of seven members, named Gel1p to Gel7p (Glucan elongase protein). The enzymatic activity of these glucanosyltransferases has been demonstrated [13,14,15]. These enzymes internally split a β(1-3)glucan chain and transfer the newly generated reducing end to the non-reducing end of another β(1-3)glucan molecule. The generation of a new β(1-3) linkage between the acceptor and donor molecule results in the elongation of the β(1-3)glucan chain [14,15]. These proteins are attached to the plasma membrane by a Glycosyl-Phosphatidyl-Inositol (GPI) anchor and have orthologues in yeasts and other filamentous fungi, *GAS* for *Saccharomyces cerevisiae*, *PHR* for *Candida albicans*, and *EPD* for *Candida maltosa* [16]. The biochemical assays performed on purified recombinant Gas1p, Phr1p, and Phr2p showed that these proteins have a β(1,3)glucanosyltransferase activity similar to that of Gel1p [17]. In *A. fumigatus*, the mutant *Δgel1* has no phenotype compared to the parental strain, whereas the mutant *Δgel2* and the double mutant *Δgel1gel2* exhibit growth delay, abnormal conidiogenesis, and altered cell wall composition. In addition, *Δgel2* and the double mutant *Δgel1gel2* show reduced virulence in a murine model of invasive aspergillosis [18]. Moreover, Gel4p is essential for the fungal life (deletion is lethal) [19]. These data showed for the first time that the β(1,3)glucanosyltransferase activity is essential for both the morphogenesis and virulence of *A. fumigatus* [18,19]. Moreover, despite the fact that these enzymes display the same enzymatic activity, their involvement in cell wall morphogenesis is different.

To date, two other β(1,3)glucanosyltransferase activities have been characterized in *A. fumigatus* (Bgt1p and Bgt2p) [20,21]. These two proteins cleave and release laminaribiose from the reducing end of linear β(1,3)glucans and transfer the remaining glucan to the non-reducing end of another β(1,3)glucan acceptor with β(1,6) linkage to create a branched polymer. Bgt1p will transfer the donor β(1,3)glucan on the non-reducing end of the chain, whereas Bgt2p will preferentially transfer within the β(1,3)glucan chain. Previously, it has been shown that the single mutants *Δbgt1* and *Δbgt2*, as well as the double mutant *Δbgt1Δbgt2*, have no distinct phenotype in terms of growth and morphology under tested conditions [21]. Moreover, these mutants show no difference in term of sensitivity to cell wall-disrupting agents such as Congo Red (CR) or Calcofluor White (CFW) and the same amount of branching of the parietal β(1,3)glucan, compared to the parental strain. These results suggest that at least one other branching activity is present in the *A. fumigatus* cell wall. Bgt1p and Bgt2p belong to the Glycosyl-Hydrolase 17 family (GH17), which possesses three other members named Bgt3p, Scw4p, and Scw11p according to their respective homologies to the protein Bgt1p or the proteins Scw4p and Scw11p (soluble cell wall proteins) of *S. cerevisiae* [22]. In our study, we analyzed the effect of successive deletions of all the GH17 family members in *A. fumigatus*.

## 2. Materials and Methods

### 2.1. Fungal Strains and Growth Conditions

The parental strain of *A. fumigatus* used in this study is the A1163 Ku80Δ strain, which comes from a clinical isolate, named CEA17 (*pyrG* +) [23]. This strain is grown in tubes containing 2% Malt agar medium. For DNA extraction, cultures were grown in Sabouraud liquid medium (2% glucose + 1% mycopeptone). Transformations were performed on minimal medium (10 g/L glucose, 0.92 g/L ammonium tartrate, 0.52 g/L KCl, 0.52 g/L MgSO_4_·7H_2_O, 1.52 g/L KH_2_PO_4_, 1 mL/L trace element solution [24], pH adjusted to 7.0). Hygromycin B (hph) (Sigma^®^, St Louis, MO, USA) was added to transformation plates in an overlay after one night of incubation at room temperature, resulting in a final concentration of 150 µg/mL. Conidia were collected from agar slants/plates after seven days of growth at room temperature using 0.05% Tween 80 solution. The mutant strains were conserved on 2% Malt medium supplemented with 150 µg/mL of hygromycin B and stored in 30% glycerol at −80 °C.

### 2.2. RNA Isolation and Analysis of the GH17 Gene Expression by RT-PCR

Mycelium of each strain was grown for 16 h in liquid MM medium and then disrupted with 0.5 mm diameter glass beads in 500 µL, and RNA was then isolated by using the phenol-chloroform method. A first DNase treatment was carried out on an RNeasy column (Qiagen^®^, Hilden, Germany) using DnaseI (Roche^®^, Basel, Switzerland) and a second one was performed after elution of the RNA from the column with Turbo DNA-free DNAse (Ambion^®^, Foster City, CA, USA). One microgram of total RNA was reverse-transcribed using a reverse transcriptase Biorad kit (Iscript cDNA synthesis kit, Bio-Rad, Hercules, CA, USA) following the instructions of the manufacturer. Quantitative PCR assays were performed according to BioRad manufacturer’s instructions using 96-well optical plates (Thermo Scientific, Waltham, MA, USA) and an iCycler iQ^®^ Real-Time PCR Detection system (170-8740, Biorad, Hercules, CA, USA). Each run was assayed in triplicates in a total volume of 25 µL containing the DNA template at an appropriate dilution, 1× Absolute SYBR green Fluorescein (Thermo Scientific, Waltham, MA, USA), and 100 mM of each primer. The primers used were designed using the Beacon Designer 4.0 software and are shown in Table S1. PCR conditions were: 95 °C/15 min for 1cycle; 95 °C/30 s and 55 °C/30 s for 40 cycles. Amplification of one single specific target DNA was checked with a melting curve analysis (+0.5 °C ramping for 10 s, from 55 °C to 95 °C). The generated data was then analyzed using iCycler iQ^®^ Opticla Sytstem Software v3.1 (Biorad, Hercules, CA, USA). The expression ratios were normalized to TEF1 expression, and calculated according to the DDCt method [25]. To verify the absence of genomic DNA contamination, negative controls in which reverse transcriptase was omitted were used for each gene set. Three independent biological replicates were performed.

### 2.3. Construction of the Single and Multiple Mutants of the GH17 Family with the β-Rec/Six System

To conduct single and multiple gene deletion, we transformed *A. fumigatus* with a the β-Rec/six-Specific Recombination System, which is a self-excising resistance marker cassette [26]. To replace the gene of interest by this marker using homologous recombination, 1 KB of the 5′ and 3′ flanking regions of each gene was amplified by PCR from *A. fumigatus* genomic DNA using primers (Forw1/Rev1 and Forw2/Rev2) (Table S1). After amplification, 5′ and 3′ fragments were ligated on both sides of the β-Rec System in the pUC19L using the GENEART seamless cloning and assembly kit (Life Technologies, A13288, Carlsbad, CA, USA). To facilitate gene recombination, before transformation, cassettes were released from the plasmid using restriction sites located inside the flanking primer cassette (see Table S1 for restriction site). *A. fumigatus* conidia were transformed by the electroporation method described by Sanchez and Aguirre [27] with subsequent modifications. Conidia were washed three times with water. 10^9^ conidia were inoculated in 125 mL of YG medium (0.5% yeast extract, 2% glucose) and incubated at 37 °C at 300 rpm for 4 h to obtain swollen conidia. Those conidia were recovered by centrifugation and then inoculated in 12.5 mL of YG medium (1% yeast extract, 1% glucose, 20 mM Hepes, pH 8.0) and incubated for 1 h at 30 °C at 100 rpm. Conidia were centrifuged and resuspended in 1 mL of cold 1 M sorbitol. 10 µg of DNA was added to 50 µL of conidial suspension, incubated for 15 min on ice, and transferred to 0.1 cm electroporation tanks. Electroporation was performed using the Bio-Rad gene pulser (Gene Pulser Xcell, Bio-Rad, Hercules, CA, USA) with the following parameters: voltage, 1 kV; capacitance, 25 microfarads; and resistance, 400 Ω. After transformation, 1 mL of cold YG medium was added to the tank, and conidia were transferred to a 10 mL sterile tube and incubated on ice for 15 min. Tubes were incubated at 30 °C at 100 rpm for 1 h and 30 min. Conidia were plated on minimal medium (500 µL/9 cm diameter Petri dish) and incubated at 20 °C overnight. Hygromycin (150 µg/mL) were added in a 10 mL overlay of minimal medium 0.7% agarose, to allow transformants selection, and plates were incubated at 37 °C for two days. After each deletion step, proper integration of the deletion cassette was verified by PCR and then by Southern Blot (Figure S1). The *A. fumigatus* single and multiple mutants constructed in this study are listed in Table S2.

### 2.4. Growth, Sporulation, Germination, Viability, and Morphology of the Mutant Strains

Mycelial growth was tested on different agar media (Malt 2%, Roswell Park Memorial Institute medium (RPMI), minimal medium (MM), and Glucose 3%-Yeast Extract 1% (YG)) at 37 °C and 50 °C, and the diameter of the colony was measured at different time points (24 h, 48 h, 72 h, and 96 h) after inoculation of 3 µL of a conidia solution at 5 × 10^6^ conidia/mL (c/mL). For the liquid condition: 10^6^ conidia were inoculated in 50 mL of liquid medium (3% glucose + 1% yeast extract) at 37 °C ,150 rpm. In order to calculate the dry weight of the fungus at each time point (24 h, 48 h, and 72 h), the mycelium was recovered on a filter (Macherey Nagel^®^ (Hœrdt, France): 0.55 mm), washed with water, dried at 80 °C for 24 h, and weighed.

The conidiation rates were estimated following the inoculation of 10^5^ conidia onto three tubes containing 2% Malt agar (10 mL/tube). After one week of growth at 37 °C, conidia were recovered with 5 mL of an aqueous 0.05% Tween 20 solution and the absorbance of the solution was measured at 600 nm (DO600 nm = 0.620 ≈ 2 × 10^7^ conidia/mL). Conidial germination was followed microscopically every 30 min after 4 h of growth at 37 °C (inoculation of 5 µL of conidia at 10^6^ cell/mL on a glass slide with 2% agar Malt medium). For the viability assays, conidia on agar slants were stored at 37 °C for two months, conidial suspensions were then recovered in 0.05% Tween 80 water, and the viability of conidia was evaluated following dilutions and plating them on 2% malt medium. The sensitivity to Congo Red^®^ and Calcofluor-White^®^ (CR: C6277 and CFW: F3543, Sigma^®^, St Louis, MO, USA) was estimated on minimal agar medium containing different concentrations of drugs (CR: 0 to 300 µg/mL and CFW: 0 to 200 µg/mL). A total of 3 µL of a suspension of conidia at 5 × 10^6^ cell/mL was inoculated on plates and incubated at 37 °C, and growth was measured at different times.

### 2.5. Carbohydrate Analysis of the Cell Wall Fractions

After 24 h of growth at 37 °C in liquid medium YG (50 mL) containing 5 × 10^7^ conidia (each strain is cultivated in triplicates), the mycelium was filtered on Buchner with two filter papers (Macherrey Nagel^®^: 0.55 mm). The resulting mycelial was resuspended in a solution of 200 mM Tris-HCl pH 8 containing glass beads (0.75 to 1 mm diameter) and disrupted in a FastPrep^®^ (MP Biomedicals, Santa Ana, CA, USA),two times 1 min at level 4, and centrifuged for 5 min at 4000× *g*, washed with water and lyophilized. Then, the cell wall was prepared as described by Gastebois et al., 2013 [28]. The neutral hexoses of the parietal fractions were estimated by the phenol sulfuric method [29]. The hexosamines have been identified and quantified by high performance liquid chromatography (HPLC), after being hydrolysed with 500 µL of 6 N·HCl for 6 h at 100 °C, as described by Johnson et al., in 1971 [30]. Branching level of β(1,3)glucan from the cell wall alkali-insoluble fraction was estimated by HPLC after laminarinase-A digestion. Prior to LamA digestion, 5 mg of AI fraction was treated by 100 mM *meta-*IO_4_Na (1 mL) at room temperature in the dark for three days. After the addition of 20 µL of glycerol for neutralization of the excess of periodate, the AI fraction was sequentially washed with water, reduced in the presence of 10 mg/mL BH_4_Na overnight, neutralized by the addition of acetic acid, washed with water, treated with 10% acetic acid at 100 °C for 1 h, and washed with water. Enzymatic digestion and the HPLC procedure have been previously described [21].

### 2.6. Phylogenetic Analysis 

GH17 protein sequences were downloaded from the PubMed website and used for generating the alignment using MUSCLE v3.8.31^1^ with default parameters. The software trimAl v3^2^ (Centre for Genomic Regulation (CRG), Barcelona, Spain) was then used to trim the alignments generated by MUSCLE (version 3.5, by Robert C. Edgar), with –gt 0.9 (for fraction of sequences with a gap allowed) and –cons 60 (for minimum percentage of the positions in the original alignment to conserve) options. We used ProTest v2.4^3^ (Free Software Foundation (FSF) Boston, MA, USA) to choose the best protein substitution model, namely LG+I+G+F. Finally, maximum likelihood analyses were conducted with PhyML v3.0.1^4^ (FSF, Boston, MA, USA) to reconstruct phylogenetic trees; support for the branches were determined from a bootstrap analysis of 100 resampled datasets.

### 2.7. Statistical Analyses

Results were statistically analyzed by an ANOVA two-way test with a Dunnet’s post-test for conidiation, growth, and survival experiment, using GraphPad Prism 6.0 software (GraphPad Software, La Jolla, CA, USA), with a *p* value below 0.05 considered as significant: (ns: not significant) (*p* < 0.05 *), (*p* < 0.01 **), (*p* < 0.001 ***), and (*p* < 0.0001 ****).

## 3. Results

### 3.1. Molecular Characterization of the GH17 Family of A. fumigatus

The Glycosyl-Hydrolase 17 family of *A. fumigatus* contains five members: Bgt1p, Bgt2p, Bgt3p, Scw4p, and Scw11p (AFUA_1G11460, AFUA_3G00270, AFUA_5G08780, AFUA_6G12380, AFUA_8G05610, respectively [31]). Characteristics and size of the proteins are shown in Table 1. Except Bgt3p, all family members contain a secretory signal peptide at the N-terminus identified by the SignalP predictor [32]. Only Bgt2p has been identified as a GPI anchored protein (BigPI and PredGPI websites) [33,34]. The percentage of identity between the members of the *A. fumigatus* GH17 family is low and varies between 9% and 28% (Table S3).

A comparative analysis of the GH17 family of *A. fumigatus* (Af), *A. nidulans* (An), *S. cerevisiae* (Sc), and *C. albicans* (Ca) shows that two putative catalytic sites identified during the study of Bgt1p and Bgt2p are conserved between all proteins (glutamate residues in position 128 and 219 of Bgt1p) (Figure S2). As shown in Figure S3, members of the GH17 family can be assigned to five distinct groups according to their evolutionary relationships based on a phylogenetic analysis with the Maximum likelihood method: Group I comprises *Af_Bgt1p* and its homologues, *Sc_Bgl2p*, and *Ca_Bgl2p*. Group II includes *Af_Bgt2p* and An_Eg1Cp with 65% of identity, both of which are GPI anchored proteins. Group III comprises *Sc_Scw4p*, *Sc_Scw10p*, *Ca_Scw4p*, and *Af_Scw4p* proteins. Group IV comprises the Scw11p protein of the three species, and finally, group V comprises only the *Af_Bgt3p* protein.

### 3.2. Gene Expression Level of the GH17 Family during Growth

**Figure 1:**
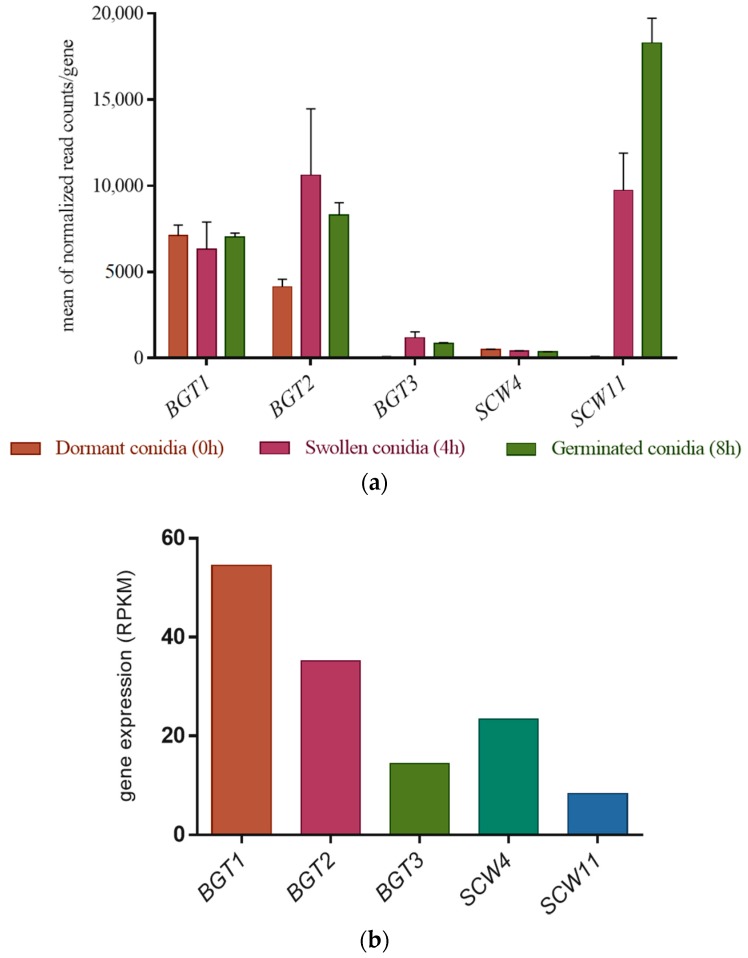
(**a**) Gene expression profile of the GH17 family genes in dormant (T 0 h), swollen (T 4 h), and germinated conidia (T 8 h) in Glucose 3%/YE 1% media, in the parental strain. The data are extracted from RNAseq analysis data [35]. The errors bars represent standard deviation from the mean values of three different experiments; (**b**) Gene expression level of the GH17 genes in the mycelium during conidiation of the parental strain (WT), values are taken from the RNA-seq data analysis of Valsecchi et al., 2017 [36]. For this RNA-sequencing experiment, the fungus was grown for seven days on malt agar at 37 °C at room temperature.

The gene expression levels of the GH17 family have been investigated in dormant, swollen, and germinated conidia by RNA sequencing analysis [35]. *BGT3* and *SCW4* remain weakly expressed during the three phases. On the other hand, *BGT1* and *BGT2* have a much stronger and relatively constant expression during the 8 h of growth. Finally, the gene expression of *SCW11* has considerably increased during conidial germination (Figure 1a).

The gene expression level of the GH17 family has also been investigated during sporulation (Figure 1b). The GH17 family is differentially expressed during conidiation, during which time *BGT1* and *BGT2* remain highly expressed. In contrast to the early developmental stages, *BGT3* and *SCW4* are more highly expressed than *SCW11* (Figure 1b).

### 3.3. Phenotypic Analyses of the Single and Multiple Mutants

#### 3.3.1. Conidiation, Viability, and Morphology of the Mutants

**Figure 2:**
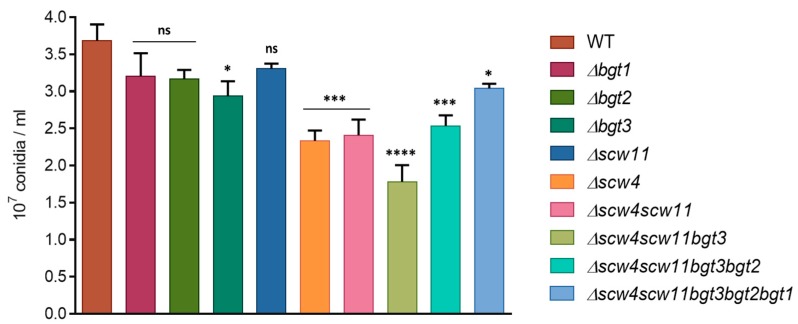
Conidiation of the GH17 mutant was measured after one week of growth at 37 °C. Values given were the mean of at least three replicates ± standard error.

To analyze the function of *A. fumigatus* GH17 proteins, simple and multiple gene deletion were undertaken. Conidiation of the mutants was determined after one week of culture at 37 °C on 2% Malt agar medium (Figure 2). A decrease of the conidiation has been observed for the single mutants *Δbgt3* (20% ± 5.4) and *Δscw4* (36% ± 3.5) compared to the parental strain. In contrast, *Δbgt1*, *Δbgt2*, and *Δscw11* single mutants sporulated like the parental strain. The triple mutant *Δscw4scw11bgt3* showed decreased conidiation by 52% ± 5.9. However, additional deletions of *BGT2* and *BGT1* resulting in the quadruple and quintuple mutants slightly increased sporulation rates.

**Figure 3:**
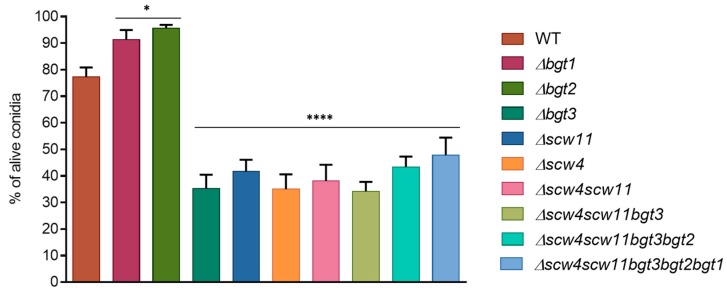
Conidial survival of different GH17 mutants after two months storage at 37 °C (WT: parental strain). Experiments were performed in triplicate.

The long-term conidial survival has been investigated after one and two months of storage at 37 °C. Conidial mortality was estimated by counting the cells able or not able to germ after this period. After one month, we did not observe significant differences. However, after two months, conidial survival decreased for all the mutants between 34% ± 3.6 and 47% ± 3.9 compared to the parental strain, with the exception of *Δbgt1 and Δbgt2* mutants (Figure 3). These results showed that the long-term viability of conidia is altered by the deletion of the *SCW4*, *SCW11*, and *BGT3* genes. In *Δbgt1* and *Δbgt2* mutants, long-term survival was improved (91% ± 3.6 and 95% ± 1.2 vs 77% ± 3.6 for the parental strain). Germination rates of the mutants were measured on 2% Malt agar medium at 37 °C. No significant difference compared to the parental strain has been observed here. 

**Figure 4:**
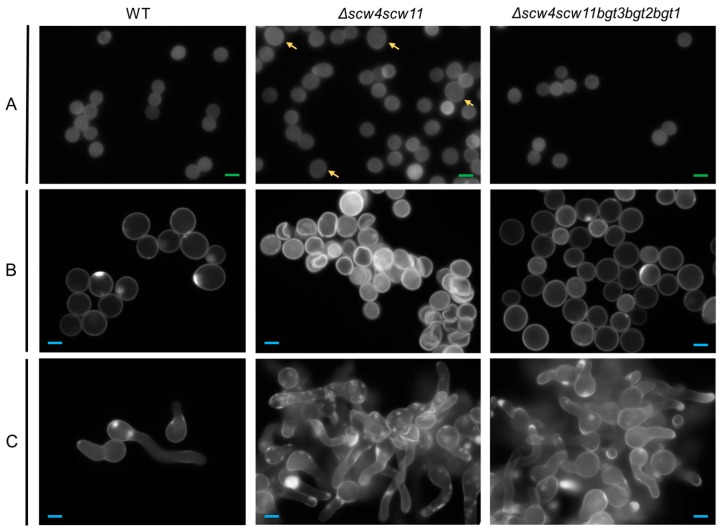
Morphology of the dormant (**A**) 0 h; swollen (**B**) 4 h; and germinated (**C**) 8 h conidia of the parental strain, and *Δscw4Δscw11* and Δ*scw4scw11bgt3bgt2bgt1* mutants using fluorescence microscopy (×63) with UV light and Calcofluor white^®^ staining, in 3% Glucose medium +1% yeast extract. Green scale bars (2 µm in panel A), blue scale bars (6 µm in B and C), and yellow arrows highlight dormant conidia with abnormal size.

Microscopic observations of conidia during germination revealed morphological changes. In *Δscw4* and *Δscw11* single mutants, as well as in the *Δscw4scw11* double mutant and the *Δscw4scw11bgt3* triple mutant, 10% of the dormant conidia showed a larger size (Figure 4). In the swollen stage (4 h) and in germinated conidia (8 h) (Figure 4B,C), 15% of the cells showed abnormal shapes: conidia clumped together or shriveled but they were still able to germinate. Moreover, following the appearance of a germ tube, we observed irregular CFW deposits (Figure 4C). In contrast, the *Δbgt1*, *Δbgt2*, *Δbgt3*, *Δscw4scw11bgt3bgt2* (*Δ4*), and *Δscw4scw11bgt3bgt2bgt1* (*Δ5*) mutants were similar to the parental strain.

#### 3.3.2. Mycelia Defects

**Figure 5:**
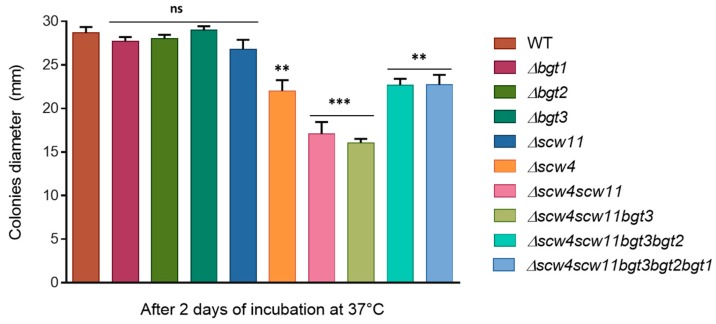
Growth kinetics of the parental strain and the GH17 mutants on RPMI agar medium at 37 °C. The diameter of the colonies was measured after 48 h. Values are mean ± SD of three independent determinations. WT, parental strain.

The growth kinetics of the mutants have been investigated. We did not observe any difference in term of biomass in liquid medium (YG). In contrast, on plates, regardless of media (RPMI, MM, YG), single mutants Δ*bgt1*, Δ*bgt2*, Δ*bgt3*, and Δ*scw11* showed similar growth to the parental strain at different culture times (24 h, 48 h, 72 h, and 96 h) (Figure 5). *Δscw4* showed a slight growth decrease (23% ± 4). In the *Δscw4scw11* double mutant, the growth decrease was more pronounced (40% ± 4, RPMI 48 h) and increased again in the triple mutant *Δscw4scw11bgt3* (44% ± 2, RPMI 48 h) (Figure 5). All these results showed that the deletion of the *SCW4* and *SCW11* genes has an additive effect on the growth rate of *A. fumigatus*. On the other hand, like for the conidiation, after deletion of the *BGT2* and *BGT1* genes, in the quadruple and the quintuple mutant, respectively, growth decrease is less pronounced than in the triple mutant (*Δ4*: 20% ± 2.5 delay and *Δ5*: 20% ± 4 RPMI 48 h), suggesting that Scw4p/Scw11p/Bgt3p proteins and Bgt1p/Bgt2p proteins might have antagonistic activities in vivo. The results obtained were similar for all the media tested and the addition of 1M of sorbitol did not restore the parental growth rate in *Δscw4scw11* and *Δscw4scw11bgt3*, suggesting that the defect is not due to an osmotic pressure imbalance.

We also analyzed the influence of the *SCW* deletion on *BGT* expression by real-time qPCR. GH17 gene expression in the vegetative mycelium of the *Δscw4*, *Δscw11*, and *Δscw4scw11* mutants and the parental strain shows no significant difference of *BGT1*, *BGT2*, and *BGT3* expression in those mutants compared to the parental strain (Figure S4). In contrast, in the *Δscw4* mutant, *SCW11* was significantly upregulated by 2.5-fold, indicating that the lack of *SCW4* tends to be compensated by *SCW11* overexpression. Like the cumulative effect found on the growth rate, this result suggests that Scw4p and Scw11p can have a similar function.

#### 3.3.3. Sensitivity to Cell Wall Disturbing Agents 

**Figure 6:**
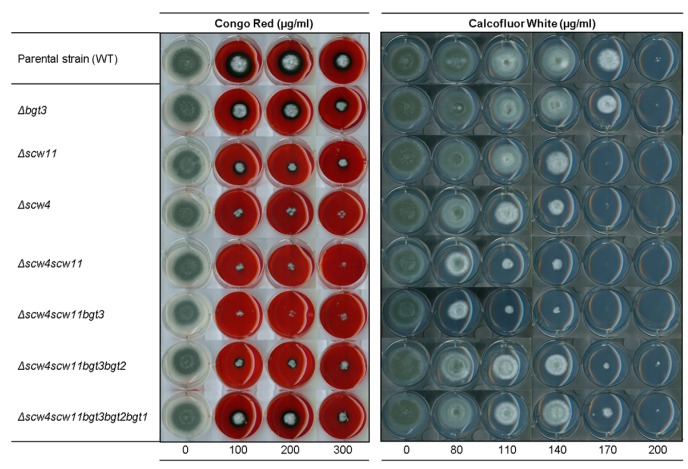
Sensitivity of the family GH17 deletion mutants to cell wall perturbing agents congo red (CR) and calcofluor white (CFW) after 72 h growth at 37 °C on MM agar media (1.5 × 10^6^ conidia were spotted). Experiments were performed in triplicate.

Sensitivity to molecules known to interfere with cell wall assembly, such as Calcofluor-White^®^ (CFW) and Congo Red (CR), has been studied. The single mutants *Δbgt1* and *Δbgt2* and the double *Δbgt1bgt2* mutant did not show increased sensitivity to CR and CFW [21], as well as the *Δbgt3* single mutant; whereas the *Δscw4* and *Δscw11* single mutants showed an increased sensitivity (Figure 6). The phenotype was more pronounced for the double mutant *Δscw4scw11*, as well as for the *Δscw4scw11bgt3* triple mutant. In contrast, in the quadruple and quintuple mutants, the sensitivity to CR and CFW is lower than in *Δscw4scw11* and *Δscw4scw11bgt3* mutants and tends towards a parental phenotype.

### 3.4. Analysis of the Cell Wall Polysaccharides

**Figure 7:**
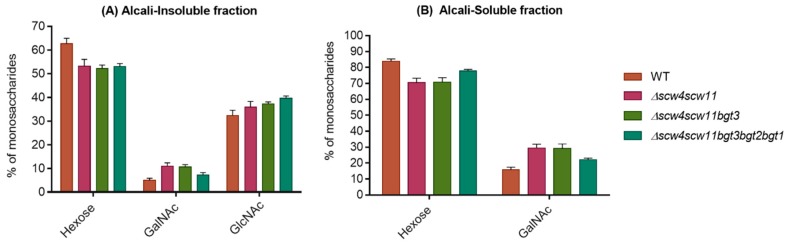
Percentage of hexose, *N*-acetylgalactosamine (GalNAc), and *N*-acetylglucosamine (GlcNAc) in the alkali-insoluble (**A**) and alkali-soluble fraction (**B**) of the cell wall of the double, triple, and quintuple mutants of the GH17 family. Values are mean ± SD of three independent determinations. WT, parental strain.

Mycelial cell wall monosaccharide compositions of the GH17 deletion strains are shown in the Figure 7. No difference in cell wall composition has previously been observed in the double *Δbgt1bgt2* mutant [21]. A decrease in hexose (glucan) levels has been observed in the AI and AS fractions for the *Δscw4scw11*, *Δscw4scw11bgt3*, and the *Δscw4scw11bgt3bgt2bgt1* quintuple mutants. This reduction is compensated by an increase of GlcNAc (chitin) in the AI and an increase of GalNAc (GAG) in both fractions. The β(1,6) branching point of the β(1,3)glucan in the GH17 cell wall mutants has been investigated. The percentage of trisaccharides β(1,3/1,6)glucan was performed by HPLC from the alkali-insoluble fractions of the double, triple, and quintuple mutants. In these three mutants, no significant decrease in the β(1,6) bonds of β(1,3)glucan has been observed.

## 4. Discussion

For the first time in filamentous fungi, the role of the complete GH17 family has been investigated. In *A. fumigatus*, five genes are present in this family of GH: *BGT1*, *BGT2*, *BGT3*, *SCW4*, and *SCW11*. In order to understand the role of these proteins in cell wall remodeling, single and multiple mutants were constructed and their phenotypes suggested that these genes have opposite functions.

Except for a beneficial effect on conidial survival, no phenotypic differences were found in the single mutants Δ*bgt1* and Δ*bgt2*, as well as in the double mutant Δ*bgt1bgt2*, as already described in previous studies [20,21]. In contrast, Δ*scw4* and Δ*scw11* single mutants, *Δscw4scw11* double mutant, and *Δscw4scw11bgt3* triple mutant showed several phenotypic defects: a decrease in conidiation associated with variable conidia size, as well as a high conidial mortality. During germination, conidia are deformed and present CFW labeling patches, suggesting cell wall embrittlement in these mutants. In addition, a reduced growth and an increased sensitivity to agents disturbing the parietal organization have been observed. Moreover, these phenotypes are reinforced after the deletion of the *SCW4*, *SCW11*, and *BGT3* genes all together, suggesting that these proteins could act synergistically. Our results showed the importance of Scw11p, Scw4p, and Bgt3p in the formation of the conidial and mycelial cell wall. Similar phenotypes were observed in the double mutant Δ*scw4scw10* of *S. cerevisiae*, with respect to CR and CFW sensitivity, survival, and cellular shape [22,37,38].

Deletion of *SCW4* and *SCW11* also induced an alteration of the parietal composition with a decrease in glucan (hexose) content compensated by an increase in chitin (GlcNAc) and GAG (GalNAc), which is a typical compensatory mechanism of cell wall mutants (for example, found in the Δ*gel2*, Δ*fks1*, and Δ*ags* mutants) [18,39,40]. These results suggest that these enzymes are involved in cell wall organization. Similar observations have been made in *S. cerevisiae*. Strains lacking *SCW4* and *SCW10* showed a decrease in the glucan amount with an increase of chitin in the alkali-insoluble cell wall fraction, suggesting that these proteins have a role in cell wall morphogenesis [22].

Surprisingly, multiple deletions comprising the *BGT1* and *BGT2* (quadruple and quintuple mutants) lead to an almost restored phenotype suggesting an antagonistic and distinct function of these two proteins with respect to the other three members of the GH17 family. These results are similar to those obtained in *S. cerevisiae* after the deletion of *BGL2* in the *Δscw4scw10* background, which also abolished most of the phenotypes of the double mutant. Furthermore, overexpression of Scw10p in *Δbgl2* drastically increased the strain’s CFW sensitivity [20]. It has also been reported that overexpression of the Bgl2p protein in *S. cerevisiae* is harmful for cell viability and appears to be lethal [41].

We investigated the gene expression level of the GH17 family during different growth stages: germination, vegetative growth, and sporulation. Depending on the growth stage, the GH17 members were differentially expressed. Even if *BGT1* and *BGT2* genes are the most highly expressed genes at every stage, no strong phenotype was associated with their deletion. During conidiation, *SCW4* and *BGT3* expression was higher than *SCW11* expression, which is correlated with the lack of a phenotype observed for the single *Δscw11* mutant. In contrast, in the vegetative mycelium of the *SCW* multiple mutants, we did not observe any variation of gene expression level for the GH17 members. Only *SCW11* was significantly upregulated in the *Δscw4* mutant, suggesting that *SCW11* can compensate *SCW4* expression and that the corresponding proteins have a similar function.

The sequence comparison of the GH17 proteins of *A. fumigatus*, *S. cerevisiae*, and *C. albicans* showed that the two glutamic acids corresponding to the putative catalytic sites are conserved between all the proteins, suggesting conserved enzymatic activities between them. However, this family can be subdivided into five subgroups, and the homologies between the subgroups are low (9 to 28% of identity). In *S. cerevisiae*, it has been shown that punctual mutation of the predicted catalytic residues (Glu326 and Glu380) in Scw10p abolished the in vivo function [13].

Interestingly, transglycosidase activities were identified in groups I and II. Af_Bgt1p and Bgt2p are able to cleave a disaccharide from the reducing end of the β(1,3)glucan polymer and then Bgt1p will transfer the remaining chain to the non-reducing end of a second β(1,3)glucan chain via a β(1,6) linkage, whereas Bgt2p will preferentially transfer within the β(1,3)glucan chain. In the cell wall of *Δscw4scw11*, *Δscw4scw11bgt3*, and *Δscw4scw11bgt3bgt2bgt1*, we did not observe a significant decrease of the β(1,6) branching point, which is in agreement with the recent data published by Aimanianda et al., in 2016 [42]. Indeed, in this study, it has been shown that branched β(1,3)glucan in the yeast *S. cerevisiae* occurs due to the cooperative activity of two glycosyltransferases, Gas1p and Bgl2p. Gas1p was responsible for 75% of the branching.

In Group III and IV, no enzymatic activity has been demonstrated for Scw4p or Scw11p proteins in *S. cerevisiae*. An *A. fumigatus* recombinant protein Scw11p has also been produced in *Pichia pastoris*, but its enzymatic analysis did not reveal any glucanosyltransferase or glucanase activity. Possibly, unlike Bgt1p and Bgt2p proteins for which we showed enzymatic activity, Scw11p may require additional factors to be active.

Nevertheless, these proteins are not closely related to Bgt1p and Bgt2p. The phenotype of the *SCW* mutants are reminiscent of the phenotype observed in other families like chitinases and β(1,3)glucanases, which have been found to be important for cell separation [43]. In *A. fumigatus,* endo β(1,3)glucanases of the GH16 and GH81 families are involved in the conidial chain separation [30] and in *S. cerevisiae*, the deletion of *SCW11* showed a separation defect between daughter cells and mother cells [44]. All of these results could suggest that Scw4p and Scw11p of *A. fumigatus* may act as β(1,3)glucanases and not as transglycosidase.

## Figures and Tables

**Table 1:** Characteristics and size of the GH17 proteins of *A. fumigatus*.

Proteins	Size (aa)	Signal Peptide	GPI Anchor	AFUA Gene Number
Bgt1p	305	1-18	NO	AFUA_1G11460
Bgt2p	446	1-18	Yes (Ω site: 423)	AFUA_3G00270
Bgt3p	688	No	No	AFUA_5G08780
Scw4p	369	1-18	No	AFUA_6G12380
Scw11p	565	1-18	No	AFUA_8G05610

## Supplementary Materials

The following are available online at http://www.mdpi.com/2309-608X/4/1/18/s1.
