# Supplementary material for: Members of Glycosyl-Hydrolase Family 17 of A. fumigatus Differentially Affect Morphogenesis
Source: J Fungi (Basel). 2018 Jan 30;4(1):18. doi: 10.3390/jof4010018 (PMC5872321; doi:10.3390/jof4010018)
Supplement: Supplementary file 1 [file jof-04-00018-s001.pdf]

Members of Glycosyl-Hydrolase family 17 of *A. fumigatus* differentially affect morphogenesis

Nicolas Millet, Jean-Paul Latgé and Isabelle Mouyna *

Aspergillus Unit, Institut Pasteur, 25 rue du Docteur Roux, 75015 Paris, France; nicolas.millet@pasteur.fr (N.M.); jplatge@pasteur.fr (J.-P.L.)

* Correspondence: imouyna@pasteur.fr; Tel.: +33-145688225; Fax: +33-140613419

Table S1. Primers used in the present study.

Primers	Sequences	Restriction site
BGT1_forw1	AATTCGAGCTCGGTACGATATCTAATGAGCCCTGAAGGCAGA	*Eco*RV
BGT1_rev1	GGACCTGAGTGATGCGGGGCAATGTTAGCAACCTA	
BGT1_forw2	TGGTCCATCTAGTGCCCGAAGTGCTCTACCGAAAG	
BGT1_rev2	GCCAAGCTTGCATGCCGATATCACCAAATCCCATCAAATCCA	*Eco*RV
BGT2_forw1	AATTCGAGCTCGGTACTGCGCACTCGGAGCAATCAAATCCAT	*Fsp*I
BGT2_rev1	GGACCTGAGTGATGCTGGAAGTACGGGTAGCCATC	
BGT2_forw2	TGGTCCATCTAGTGCCCAAGACCTACTGGGACGAG	
BGT2_rev2	GCCAAGCTTGCATGCCTGCGCAATCGGGGCTATTAGCAGGAC	*Fsp*I
BGT3_forw1	AATTCGAGCTCGGTACTGCGCACGGATCAATTGACTCGACCT	*Fsp*I
BGT3_rev1	GGACCTGAGTGATGCAGCAATGACAGCGGAGAACT	
BGT3_forw2	TGGTCCATCTAGTGCTTCGATAGCTCTTGGCACCT	
BGT3_rev2	GCCAAGCTTGCATGCCTGCGCAGTCGATTGCTGCGATCAGTA	*Fsp*I
SCW4_forw1	AATTCGAGCTCGGTACCCCGGGGGAGACCGCTTTCTCAAGTG	*Sma*I
SCW4_rev1	GGACCTGAGTGATGCCGTTGAATCTTCTGGGCAAT	
SCW4_forw2	TGGTCCATCTAGTGCGCTCCTGTGGAGGTTAGCTG	
SCW4_rev2	GCCAAGCTTGCATGCCCCCGGGGGAGTCTGGTACTCCCACCA	*Sma*I
SCW11_forw1	AATTCGAGCTCGGTACGATATCTATTGCCTCCCAGTCCAAAG	*Eco*RV
SCW11_rev1	GGACCTGAGTGATGCGATCTGATTCGGTCGCAACT	
SCW11_forw2	TGGTCCATCTAGTGCAACATCCACCCCTTCTTCAA	
SCW11_rev2	GCCAAGCTTGCATGCCGATATCAGGATAACTGCCGCGATGT	*Eco*RV
BGT1qa	AGAATCCAGCAGGTCCAGAAGG	
BGT1qb	CCGTGCCATCAGCGAACTTG	
BGT2qa	CGGTATCGTCGGCAGCACTC	
BGT2qb	GGCGGCGGAGGAAGAAGAAG	
BGT3qa	ATCCTGGCTTTCATCATCATTGGG	
BGT3qb	CTTCGGTGGCTGTTTGTGTGG	
SCW4qa	TCGCTTCGGCATCACCTACTC	
SCW4qb	TGCTCAATGTCCTGGTTTATCTGG	
SCW11qa	TCGTCGTCGGTAATGAGGCTATC	
SCW11qb	GGCGGACTTGGCAGAGGAG	
TEFqa	CCATGTGTGTCGAGTCCTTC	
TEFqb	GAACGTACAGCAACAGTCTGG	

Table S2. Single and multiple deleted strains used and constructed in this study.

Strain Names	Genotype	Source
Parental strain (WT)	A1163$^{ku80\Delta}$	Da Silva Ferreira *et al.*, 2006
Δbgt1	A1163$^{ku80\Delta}$ Δbgt1::hygroR	Gastebois A. *et al.*, 2010
Δbgt2	A1163$^{ku80\Delta}$ Δbgt2::hygroR	Gastebois A. *et al.*, 2010
Δbgt3	A1163$^{ku80\Delta}$ Δbgt3::six-β-rec-hygroR-six	This Study
Δscw4	A1163$^{ku80\Delta}$ Δscw4::six-β-rec-hygroR-six	This Study
Δscw11	A1163$^{ku80\Delta}$ Δscw11::six-β-rec-hygroR-six	This Study
Δscw4Δscw11 (Δ2)	A1163$^{ku80\Delta}$ Δscw4::six/Δscw11::six-β-rec-hygroR-six	This Study
Δscw4Δscw11Δbgt3 (Δ3)	A1163$^{ku80\Delta}$ Δscw4::six/Δscw11::six/Δbgt3::six-β-rec-hygroR-six	This Study
Δscw4Δscw11Δbgt3Δbgt2 (Δ4)	A1163$^{ku80\Delta}$ Δscw4::six/Δscw11::six/Δbgt3::six/Δbgt2::six-β-rec-hygroR-six	This Study
Δscw4Δscw11Δbgt3Δbgt2Δbgt1 (Δ5)	A1163$^{ku80\Delta}$ ΔScw4::six/Δscw11::six/Δbgt3::six/Δbgt2::six/Δbgt1::six -β-rec-hygroR-six	This Study

Table S3. Percentages of identities between the different proteins of the *A. fumigatus* GH17 family.

	Bgt2p	Bgt3p	Scw4P	Scw11P
**Bgt1p**	26	25	20	16
**Bgt2p**		10	14	9
**Bgt3p**			12	14
**Scw4P**				28

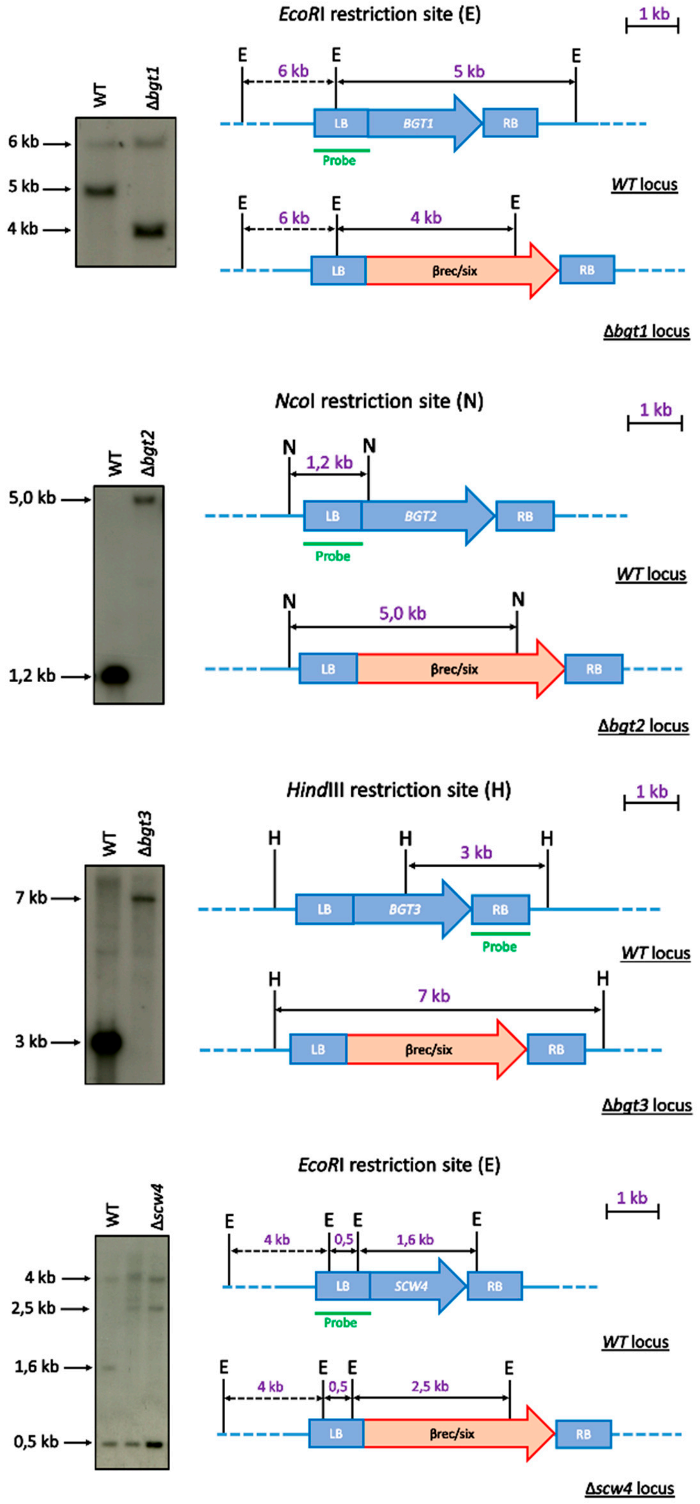

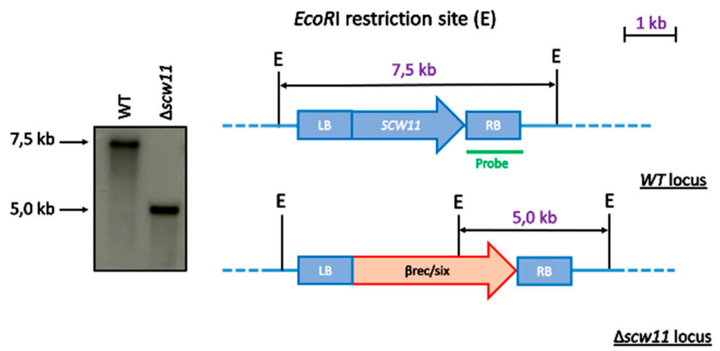

Figure S1. Construction of the deletion strains. Subsequent deletion of the GH17 genes family and verification by Southern blot.

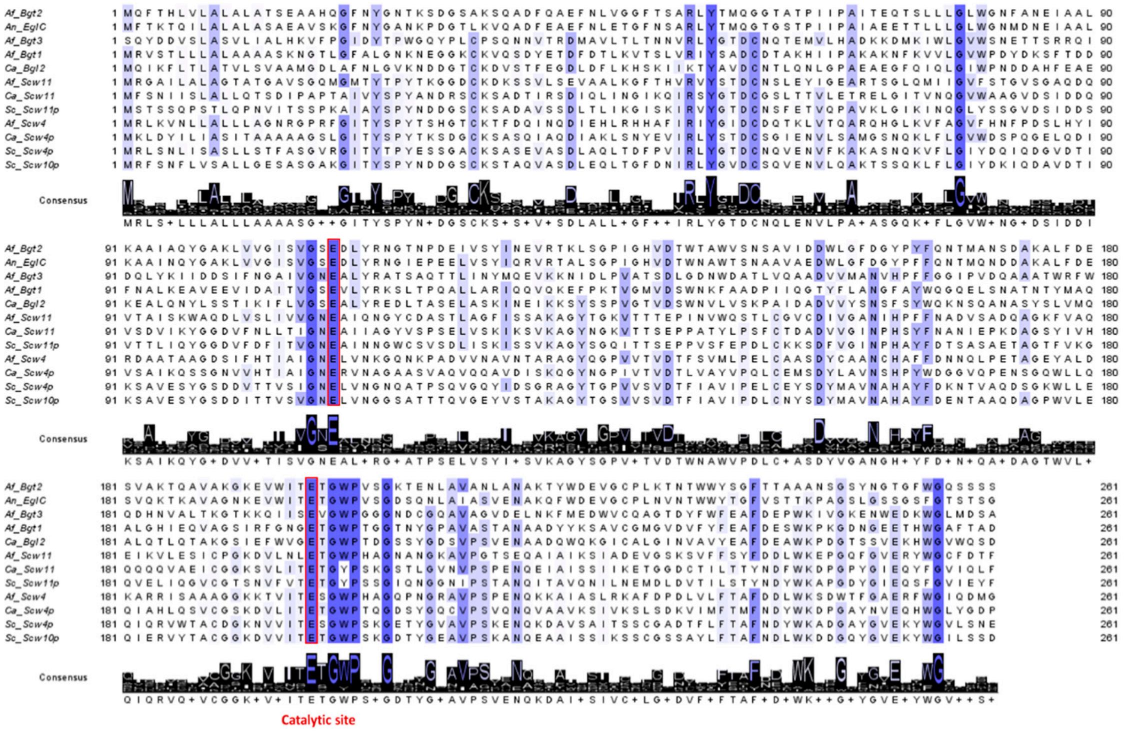

Figure S2. Protein alignment of the GH17 family of *A. fumigatus* (Af), *S. cerevisiae* (Sc), *C. albicans* (Ca), and *A. nidulans* (An). Conserved Amino acids between all proteins are highlighted in blue. Both glutamic acids, which are putative catalytic sites, are framed in red. After protein sequence alignment using MUSCLE v3.8.311, sequences were trimmed using TrimAl v3[2] to remove the spurious sequences or poorly aligned regions.

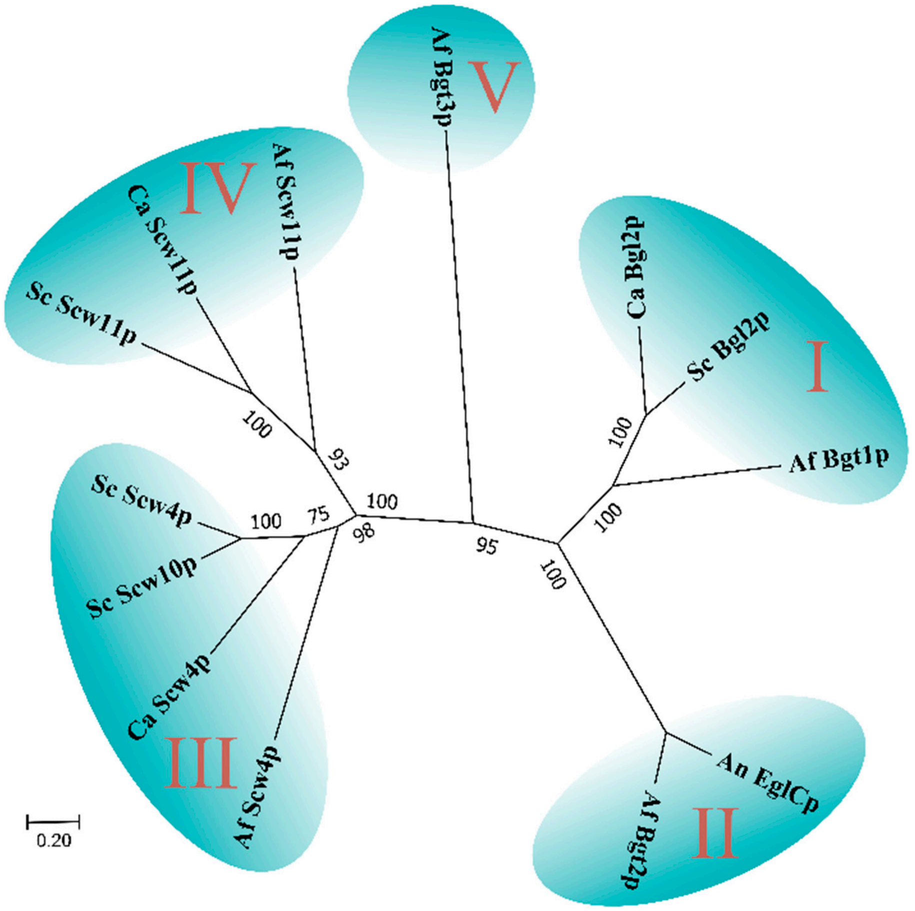

Figure S3. Non-rooted tree showing the maximum likelihood phylogenetic tree of the GH17 proteins of *A. fumigatus* (Af), *A. nidulans* (An), *S. cerevisiae* (Sc), and *C. albicans* (Ca). The protein sequences used have the following Pubmed accession numbers: For *A. fumigatus*: Bgt1p (AMO45773.1), Bgt2p (EAL86311.1), Bgt3p (Q4WUK5.1), Scw4p (XP_751116.1), and Scw11p (XP_747362.1); *A. nidulans*: (AAT90341.1); *S. cerevisiae*; Bgl2p (EGA58533), Scw10p (AJS99243.1), Scw4p (AJS06938.1), and Scw11p (AJS07159.1); *C. albicans*; Bgl2p (AOW28996.1), Scw11p (AOW29820.1), and Scw4p (AOW25936.1).

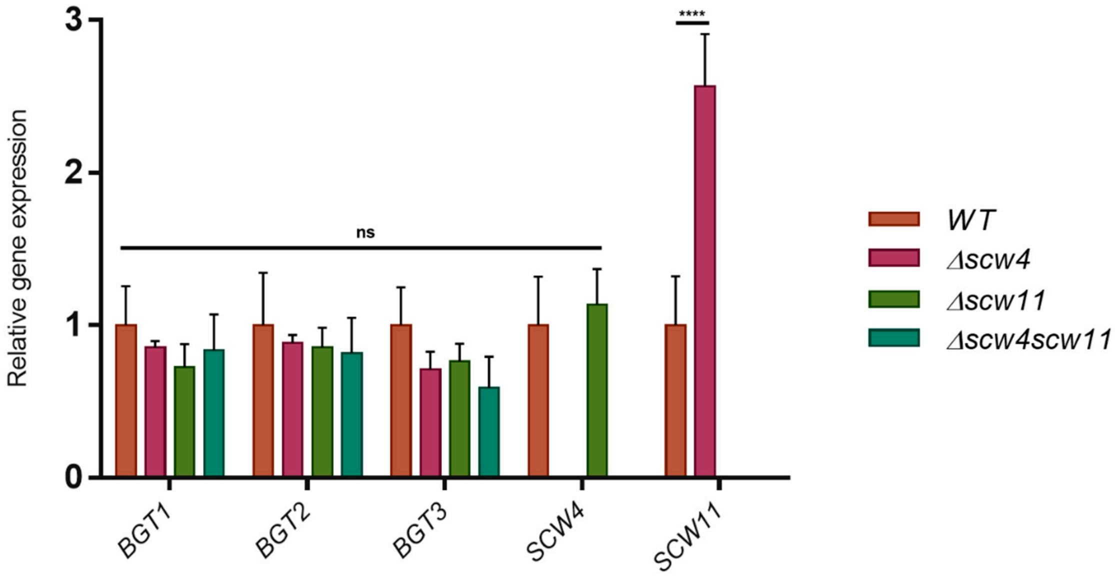

Figure S4. Gene expression of the five GH17 genes in the vegetative mycelium of the *Δscw4*, *Δscw11*, and *Δscw4scw11* mutants and the parental strains after 16 h of growth in MM liquid medium (**** statistical significance between the parental and the mutant strain, $p < 0.0001$). Gene expression was determined by real-time RT-PCR.
